# Community-led cross-sectional study of social and employment circumstances, HIV and associated factors amongst female sex workers in South Africa: study protocol

**DOI:** 10.1080/16549716.2021.1953243

**Published:** 2021-08-02

**Authors:** Minja Milovanovic, Rachel Jewkes, Kennedy Otwombe, Maya Jaffer, Kathryn Hopkins, Khuthadzo Hlongwe, Mokgadi Mathaludi, Venice Mbowane, Glenda Gray, Kristin Dunkle, Gillian Hunt, Alex Welte, Reshma Kassanjee, Nevilene Slingers, Lieve Vanleeuw, Adrian Puren, Anthony Kinghorn, Neil Martinson, Fareed Abdullah, Jenny Coetzee

**Affiliations:** a Perinatal HIV Research Unit, Faculty of Health Sciences, University of the Witwatersrand, Soweto, South Africa; b African Potential Management Consultancy, Kyalami, South Africa; c Office of the Executive Scientist, South African Medical Research Council, Pretoria, South Africa; d Gender & Health Research Unit, South African Medical Research Council, Tygerberg, South Africa; e School of Public Health, Faculty of Health Sciences, University of the Witwatersrand, Johannesburg, South Africa; f Office of the President, South African Medical Research Council, Tygerberg, South Africa; g National Institute of Communicable Diseases (NICD), Johannesburg, South Africa; h Centre for Infectious Disease Epidemiology and Research, School of Public Health and Family Medicine, University of Cape Town, Cape Town, South Africa; i South African National Department of Science and Innovation – National Research Foundation (DSI-NRF) Centre of Excellence in Epidemiological Modelling and Analysis (SACEMA), Stellenbosch University, Stellenbosch, South Africa; j Office of AIDS and TB Research, South African Medical Research Council, Pretoria, South Africa

**Keywords:** Key populations, community-centric, national survey, HIV prevalence

## Abstract

**Background:**

In South Africa, female sex workers (FSWs) are perceived to play a pivotal role in the country’s HIV epidemic. Understanding their health status and risk factors for adverse health outcomes is foundational for developing evidence-based health care for this population.

**Objective:**

Describe the methodology used to successfully implement a community-led study of social and employment circumstances, HIV and associated factors amongst FSWs in South Africa.

**Method:**

A community-centric, cross-sectional, survey of 3,005 adult FSWs was conducted (January–July 2019) on 12 Sex Work (SW) programme sites across nine provinces of South Africa. Sites had existing SW networks and support programmes providing peer education and HIV services. FSWs were involved in the study design, questionnaire development, and data collection. Questions included: demographic, sexual behaviour, HIV testing and treatment/PrEP history, and violence exposure. HIV rapid testing, viral load, CD4 count, HIV recency, and HIV drug resistance genotypic testing were undertaken. Partner organisations provided follow-up services.

**Results:**

HIV Prevalence was 61.96%, the median length of selling sex was 6 years, and inconsistent condom use was reported by 81.6% of participants, 88.4% reported childhood trauma, 46.2% reported physical or sexual abuse by an intimate partner and 57.4% by a client. More than half of participants had depression and post-traumatic stress disorder (52.7% and 54.1%, respectively).

**Conclusion:**

This is the first national survey of HIV prevalence amongst FSWs in programmes in South Africa. The data highlight the vulnerability of this population to HIV, violence and mental ill health, suggesting the need for urgent law reform. Based on the unique methodology and the successful implementation alongside study partners, the outcomes will inform tailored interventions. Our rapid rate of enrolment, low rate of screening failure and low proportion of missing data showed the feasibility and importance of community-centric research with marginalised, highly vulnerable populations.

## Background

South Africa is at the global epicentre of the HIV pandemic, and within South Africa, sex workers (SWs) have the highest HIV prevalence of any risk group [1]. One percent of the total adult female population in South Africa are female sex workers (FSWs) [2], with an estimated HIV prevalence of 40–90%, depending on their location [1,3,4]. This is 2–4 times higher than the 23% HIV prevalence recorded in women aged 15 years and older in the general population [5]. Recent evidence suggests that 40.8% of new infections may be attributed to clients of sex workers, who place both sex workers and their other partners at risk [6]. Outreach treatment programmes have enabled many with HIV to access ART, but there are worrying developments around drug resistance. A 2017 study found that 16% of HIV positive FSWs in Soweto, an area south-east of Johannesburg, had detectable HIV drug resistance (HIVDR). Among these, one quarter were ARV treatment naïve at the time of specimen collection [7]. This suggests the sexual acquisition of HIVDR, and this finding amongst FSWs in a major urban area highlights the urgency of studying HIV and HIVDR transmission dynamics amongst FSWs in South Africa more broadly.

Research on the sex worker HIV epidemic in South Africa has primarily been confined to the three main metropolitan areas: Johannesburg, Cape Town and Durban [1,4,6–9]. Interventions geared towards HIV prevention and response amongst SWs have largely focused on behaviour change (sexual risk reduction), promoting HIV testing, antiretroviral therapies (ART) and/or pre-exposure prophylaxis (PrEP) initiation, and post-violence linkage to care [10]. Exposure to violence in these populations is disproportionately high. Evidence suggests that 90% of FSWs have been exposed to either sexual or physical violence at some stage in their lifetime, with over 60% to sexual violence [11]. Violence is associated with exceptionally high levels of adverse mental health outcomes [12]. A study in Soweto found that 69% of FSWs had symptoms of major depression, while 40% had symptoms of post-traumatic stress disorder (PTSD) [13]. Violence and poor mental health both have an adverse impact on adherence to chronic medication [1,14]. Evidence from the Soweto study suggests that pathways to HIV and HIVDR intersect with both violence and poor mental health [1]. However, it is not known whether this was the case for FSWs across South Africa. To effectively develop and deliver sex work programmes, it is vital to ensure that interventions match the specific vulnerabilities experienced by FSWs and noting, in particular, that the factors driving HIVDR are not well understood.

This study was designed to offer the first national-level data on HIV prevalence, incidence, violence, mental ill health, sexual and other risks, and treatment access among FSWs in networks of programmes in South Africa. Early indications suggest that the SARS-Cov-2 pandemic has impacted healthcare service delivery, with one study suggesting an almost 50% reduction in HIV testing and ART initiation post the onset of the first COVID-19 lockdown in South Africa [15], with modelling estimates suggesting a 1.63 times increase in HIV related deaths [15]. Further data indicate the escalation of gender-based violence during South Africa’s harsh lockdown [16]. Thus, this study provides a pre-COVID-19 pandemic, comprehensive national baseline estimate of HIV, violence and mental illness amongst FSWs. In this manuscript, our objective is to describe the methodology used to implement a community-led cross-sectional study of social and employment circumstances, HIV and associated factors amongst FSWs in South Africa. The results section of this manuscript is used to further highlight the successful implementation of the described methodology.

## Methods/design

SWs in South Africa are exceptionally vulnerable for many reasons, including: on-going criminalisation of sex work [17], high levels of stigma, homophobia and gender-based violence in communities. This study focused on FSWs because funding was available for research on adolescent girls and women. Additional research is urgently needed to understand the dynamics of male, gay and trans sex workers. Working with sex workers, it was therefore important to use multiple strategies to protect participants from potentially physical, emotional, legal, health and social stigma threats during the research study.

Accessing this population posed unique and often challenging concerns, some of which were ethically complex. The use of existing programmes working with sex work communities was paramount to accessing and recruiting the population, ensuring linkage to care, capacity development, and to rapidly building trust with potential participants. South Africa has a history of implementing sex work programmes, which have predominantly been funded by PEPFAR or The Global Fund. At the peak of their funding (2013–2016), there were >30 sex work programmes across South Africa. Over time, and as HIV in key populations has garnered increasing support, these generally community-centric interventions have become increasingly HIV continuum of care (CoC) focused, with greater in-house clinical components being introduced since 2016. With funding constraints, the number of programmes has decreased, with sites centralised to high HIV transmission areas (for the general population). This, however, meant that sex workers in very rural or semi-urban areas were unlikely to have received services (or to be represented in this research study). Only one province in South Africa had no formal clinical sex work programme (the Northern Cape); however, a small outreach team worked in one district providing basic education and some commodities.

At the time of this study, sex work programmes focused on HIV CoC and PreExposure Prophylaxis (PrEP) was being rolled out across sites. All sites housed peer outreach programmes, which included outreach by SW peer educators (individuals who were or had been SWs, and then worked on a sex work project assisting with funded activities around HIV prevention) who provided: HIV testing and linkage to care, commodity distribution, community support workshops and linkage to human rights services. Outreach services were provided at the venues where sex was sold (e.g. open spaces included streets, bushes, or beaches, and indoor venues included brothels, taverns or hostels). Site peer education programmes leveraged the well-connected sex work network to mobilise sex work communities, disseminate information and access new venues/SWs.

Mapping South Africa’s nine provinces (with 52 districts) resulted in identification of operational sex work programmes (sites) and 22 districts where they operated sex work programmes. In our sample, these 22 districts were stratified by province. Most districts housed only one sex work programme. Certain districts in Gauteng and KwaZulu-Natal housed multiple programmes in order to cater for the size of the population and/or geographic distribution. In these cases, we selected a partner project based on the organisation’s research capacity and their history of community-centric work with marginalised populations. This partner was expected to work across the whole district and not just in the organisation’s area.

### Sample size calculation

The sample size was determined using the method for prevalence, where = sample size, z = z-statistic for level of confidence, = expected prevalence and = precision. The target sample size was set to achieve a precision of between 1.8% and 5% using prior prevalence estimates for HIV (39%–70%) [1,3,18]. Assuming a HIV prevalence of 50%, a precision of ±1.8% and a z-value of 1.96, we set a recruitment target of 3000 FSWs to be enrolled across all randomly selected study sites.

### Sampling study sites and participants

**Figure 1.**
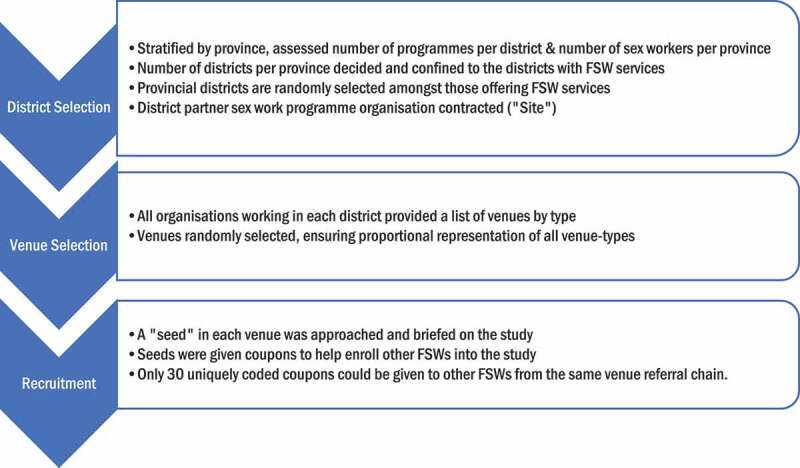
Multistage sampling procedure allowed for a close approximation of a nationally representative sample

A multi-stage sampling procedure was undertaken in order to derive a self-weighting sample stratified by province (Figure 1). The recruitment phase loosely draws on the elements of a respondent-driven sampling method for hard-to-reach populations [19].

The 22 eligible districts were stratified by province. Normally, one district was selected per province. However, in deciding the number of districts to be included per province, we reviewed the number and density of sex work programmes in each district. The 2013 sex worker size estimation per province and district [20] was used to proportionately divide up the total sample size across the provinces. We used a cut-off of 380 FSWs for our estimated provincial sample size (Table 1) to determine whether the sample for that province should be divided across more than one district (Table 2). The 380 cut-off was based upon the enrolment timeframe and resources funded per site, and allowed for greater diversity in the sample. For example, Gauteng comprises 5 districts and had eight sex work programmes, an estimated 28.7% of FSWs work across the province, and the target sample size was 854 for the province (Table 1). Considering these factors, it was decided that three districts should be included in the study. These districts were then randomly selected from the Gauteng list of districts that housed sex work programmes (Table 2). In each district, a partner sex work organisation was contracted to undertake recruitment and data collection.

**Table 1.** Number of study sites per Province and target sample size per site based upon the proportion of Sex Workers estimated to work in the province [20]

Province	Estimated FSW population	Percentages of estimated national FSW population size	Target Sample size per province	# of Districts Sampled*
Gauteng	32,824	28.5%	854	3
KwaZulu Natal	16,797	14.6%	437	2
Western Cape	14,529	12.6%	378	1
North West	10,685	9.3%	278	1
Mpumalanga	10,224	8.9%	266	1
Eastern Cape	9 917	8.6%	258	1
Limpopo	9 686	8.4%	252	1
Free State	7 264	6.3%	189	1
Northern Cape	3 382	2.9%	88	1
	**115,308**		**3 000**	**12**

**Table 2.** Districts selected per province showing the proportion of FSWs to be enrolled in each proportionate to estimated population size, target sample size and target number of venues

Province	Randomly Selected Districts, each having one partner Site for recruitment	Percentages of estimated FSW population size in Districts	Target Sample size per District	Target number of venues per District to achieve sample size
Gauteng	**City of Johannesburg Metro**	62%	528	53
Gauteng	**Ekurhuleni Metro**	20%	167	17
Gauteng	**City of Tshwane Metro**	18%	159	16
KwaZulu Natal	**Ethekwini Metro**	82%	357	36
KwaZulu Natal	**Ugu**	18%	80	8
Western Cape	**City of Cape Town Metro**	100%	378	39
North West	**Bojanala**	100%	278	28
Mpumalanga	**Ehlanzeni**	100%	266	27
Eastern Cape	**Buffalo City Metro**	100%	258	26
Limpopo	**Vhembe**	100%	252	25
Free State	**Thabo Mofutsanyane**	100%	189	19
Northern Cape	**Frances Baard**	100%	88	9
Total Target Sample			**3000**	

Each partner organisation (‘Site’) provided the study team with a list of venues (stratified by the type of venue – bush, brothel, street) where sex was known to be sold, and which they had recently mapped from across their district. In areas where multiple organisations worked across a district, all sex work programme organisations agreed to provide a list of mapped venues for inclusion in the sampling process. For example, the City of Johannesburg had four SW programmes working in specific areas. While only one organisation was contracted to undertake the study, all four contributed to the list of venues mapped across the district.

To ensure that a diverse group of FSWs were enrolled through each initial venue recruit (called a ‘seed’), it was estimated that the minimum enrolment per venue would be 10 and the maximum 30 FSWs. Based upon this, and to ensure that a representative proportion of venue-types were selected, the number of venues to be selected per site was calculated as (Table 2). Thus, for example, the City of Johannesburg had a sample size of 528, and 53 venues were selected. The final venue lists were provided for the sites, with each site-venue ascribed to a unique code (e.g. COJ-A). Lists included a randomly selected and proportional representation of each venue-type relevant to that district.

**Figure 2.**
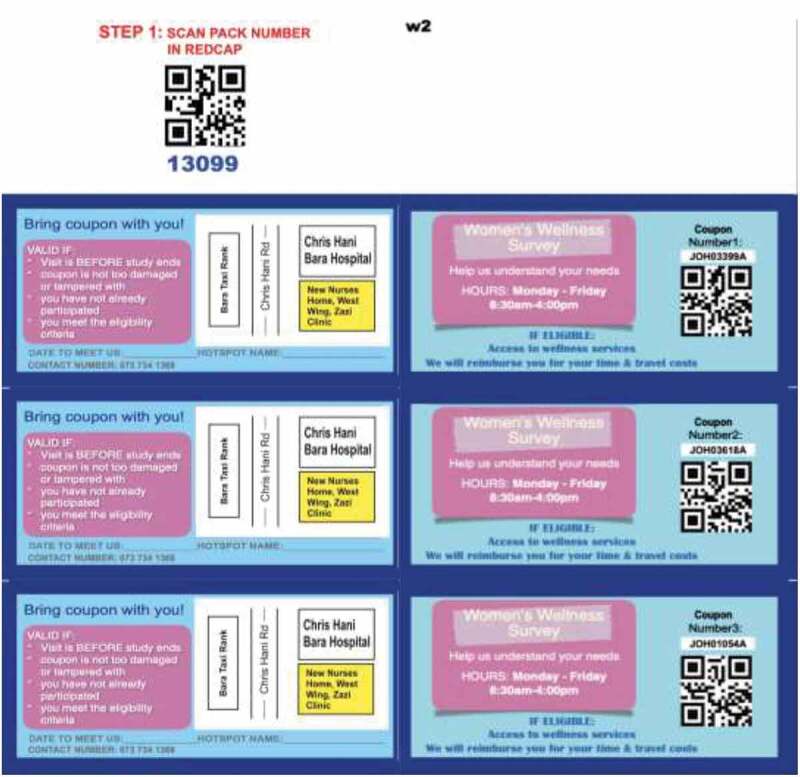
Example of study coupon

At a venue, an initial FSW recruit ‘seed’ was chosen on the day that the partner organisation’s peer education team visited the venue. The seed was briefly told about the study and invited to visit the site to hear more about the proposed research, ask questions and potentially be enrolled. FSWs who agreed were issued with a uniquely coded coupon. All coupons within the site’s enrolment chain included a study-specific site-venue code along with an additional 5-digit random coupon number (COJ 22,543 A). Thus, all coupons were uniquely coded for each district. Coupons were printed in uniquely coded packs of three (see Figure 2). Only 10 packs per venue were printed, allowing a maximum of 30 coupons per venue to be distributed. Creating coupon packs simplified coupon management at a site level. It also facilitated monitoring the chain of recruitment. This strategy supported our community-centric approach and ensured that no inter-district enrolments occurred. Using uniquely coded coupons per district, site staff could quickly ascertain if a coupon from one district had crossed over to another district to attempt enrolment. To further limit inter-district enrolment, we also collected participant contact details and other individual identifying variables that were cross-referenced in the database during the enrolment process.

Seeds and then subsequent FSWs who visited the data collection sites with a coupon were screened, consented and enrolled into the cross-sectional survey and sample collection. After data collection, each seed and her first 10 (successfully enrolled) recruits were each given a coupon pack (Figure 2) and asked to distribute a coupon to three fellow FSWs who they knew, regardless of whether these FSWs worked within the same venue or not. Recruited FSWs could then come to the site and be screened for eligibility and enrolled if successful. The average number of participants recruited from a single seed was 9–10 people. Participants were reimbursed for their time, to the value of ~15USD. The value of the reimbursement was influenced by multiple factors, including: budgetary considerations, norms established through previous studies with sex workers, study procedures to be undertaken, ethical considerations, the earning potential of SWs [1] and stakeholder input on reimbursement.

From the list of venues, a new venue-seed was only recruited under authorisation of the core team, who staggered seed recruitment to help manage the rate of enrolment at a site level and ensure that sites were not overwhelmed by potential participants wanting to enrol.

### Community-centric research development and staff training

Our study leveraged the existing infrastructure in sex work programmes to access networks of FSWs. This allowed us to build capacity within established programmes, and minimised both disruption and confusion by avoiding a ‘new’ organisation working in the area. Being embedded within existing sex work programmes also allowed the study team to work with SWs and not just study them. They were meaningfully integrated into the research teams at each site and provided invaluable input to improve data collection. By working with local organisations, which have established referral networks, we were able to ensure access to support services, ART and PrEP post the study.

Based on previous work [1], a staged approach was used to ensure community buy-in and active participation in the study. Interview guides and questionnaires were developed in collaboration with SWs and sex work peer educators. Cognitive interviews had previously been undertaken on many elements of the questionnaire to ensure the phrasing was appropriate and understood correctly. Based upon this previous work, an initial draft of the questionnaire was pre-tested among a group of FSWs and SW peer educators (current or former SWs employed by an SW programme to provide HIV and outreach services). Feedback was provided on the questionnaire. For example, economical violence was raised as a missing component, and additional locations where sex was sold were suggested. These recommendations were incorporated into the survey tool.

A preliminary workshop was used to further refine the survey tool and ensure that each of the communities within which the research would be undertaken agreed with the questionnaire’s structure and content. Feedback was sought on recruitment methodologies, input and information on venues in each district, and differences in population characteristics to allow the primary study team to plan and effectively support the implementation of the research project. Sites and community members provided guidance on where participants would feel most comfortable being interviewed, as well as logistical considerations for enrolment at each site. This was especially important in rural districts, where travelling distances may have hampered women accessing study sites. Feedback also included suggestions on the types of services SWs had or would like to have received through programmes, the time of day and days of the week when clinical services should be provided. All feedback from community members and sites was incorporated as much as possible into the study design. The sex work programmes were asked to brief the FSW community in their district as well as owners of venues about the study to help ensure access and buy-in from key stakeholders.

To become interviewers, SW peer educators were provided 40 hours of intensive training on lay psychosocial counselling, covering topics including basic counselling, trauma containment and debriefing. This strategy ensured SW peer educators were able to provide basic containment to participants as required during interviews as well as to the FSW community once they were integrated back into the sex work programme on study closeout. SW peer educators’ emotional wellbeing was supported through greater awareness of emotive triggers and the need to engage in mental healthcare. SW peer educators were taught coping skills to help themselves deal with stress and trauma (skills they can impart to the community), and SW peer educators acquired additional occupation skills for future work opportunities and the possibility of improved salary support.

A total of 25 SW peer educators were trained for the study, allowing all study interviews to be conducted by a gender matched (female) peer educator. This community-centric strategy ensured that potential participants could be easily screened to confirm they met the inclusion criteria, and that interviewers could rapidly build a rapport with participants to ensure openness during interviews. It also minimised the risk of stigma and discrimination against participants, and ensured that non-sex worker study staff were continuously sensitised to sex worker life concerns by their colleagues. It was imperative that the voices of sex workers were privileged throughout the study.

Non-sex worker project staff attended a 1-day compulsory sex work sensitisation training [21] workshop facilitated by an experienced sex worker advocacy organisation. This afforded staff the opportunity to develop greater understanding and empathy towards the experiences of sex work research participants.

Ethics and study-specific training were also provided to all staff, including SW peer educators, as was data management training using the chosen REDCap electronic database [22]. In this way, the project was able to give back to communities through upskilling of study staff in meaningful ways, and through providing counselling skills, which, post-study, could be used within the sites’ sex work programmes.

### Eligibility

For inclusion in the study, participants needed to be biologically females 18 years or older and to have voluntarily sold or transacted during sex for financial gain (not necessarily paid in cash) in the past 6 months. They also needed to work within the site’s district. However, FSW did not have to have previously accessed services or visited the SW programme to be considered for inclusion in the study. For ethical and human rights reasons, individuals who were <18 years, or who self-reported that they were current victims of human trafficking, were excluded from enrolment in this study. When a site’s study team became aware of child or victim of human trafficking, the site’s sex work programme was notified and their organisational specific protocols were implemented, including legal and psychosocial support. Screening and informed consent was undertaken by an SW peer educator.

### Data tools

#### Survey

After screening and voluntary informed consent, participants completed a 40-minute interviewer-administered survey (Table 3). Interviewers strategically sat next to participants and shared the data collection tablet between them. Thus, through a proactive and empowering interview process, participants were enabled to become part of the data collection process and to feel engaged and in control of responses being captured. This strategy was important in equalising the balance of power between researcher and subject. It also minimised eye contact and gave a common focal point, which was a useful tool when capturing difficult or intimate details of sexual behaviour or violence exposure. The questionnaire tools used as part of the survey have been previously tested [1,7,11,13]. The survey tool was translated, by study staff members, into 10 local languages based on the most commonly spoken languages across the 12 sites. Each translated tool was reviewed by multilingual study staff and a certified translation company to confirm the appropriateness of the translations to the dialects spoken by FSW in each district.

**Table 3.** Key questionnaire measures

QUESTIONNAIRE	ALPHA *	MEASURE	DETAILS
Demographic Information		PHRU demographics form	Included: Date of birth, nationality, level of education, number of dependents, age first entered into SW
HIV testing history and adherence, TB symptom screening		PHRU HIV Testing history and Tuberculosis (TB) Symptoms Screening	HIV testing behavior including reasons for testing or not testing, most recent test date, self-reported HIV status and detailed Antiretroviral Therapy (ART) and Pre-Exposure Prophylaxis (PrEP) uptake are assessed. TB symptoms screening was conducted according to the WHO guidelines [23].
Childhood Experiences	0.81	Childhood Trauma Questionnaire (short version) (CTQ) [24]	A modified version of the CTQ which measures 5 dimensions: neglect (physical and emotional) and abuse (emotional, physical and sexual), was used. We added questions on female circumcision which was highlighted during the initiation of one of the sex work programs as possibly being prevalent amongst certain ethnic groups in South Africa. These items were not scored with the CTQ scale.
Pregnancy and Children		WHO Sexual and Reproductive Health (SRH) questionnaire adapted [25]	Asked about most recent pregnancy, overall pregnancies, contraceptive use and termination of pregnancy.
Exposure to Violence		WHO violence against women [26,27] adapted for FSW	WHO Violence Against Women instrument was adapted for sex work, and assesses violence (physical, emotional and sexual) perpetrated by a primary intimate partner, clients, police and other males.
Sexual Behavior		Primary and non-primary partner questions, dry sex practices [26]. WHO sexual and reproductive healthcare questionnaire [25] as well as Stepping Stones Questionnaire [28] adapted	Adaptation of the WHO Adolescent sexual and reproductive health survey {44}, which has previously been used in a study of SW {49}. The new instrument documented knowledge, beliefs, testing behaviours, attitudes and outcomes of sexual health behaviour by SW, length of time as a SW and SW sexual practices based upon a study of male SW which indicated types of sex acts common in SW. All items on this scale were scored singularly. Certain items were summed.
Depression	0.90	CES-D 10 [29] short	Depression and suicide ideations were assessed. CES-D10 scores: >10 was considered to indicate depression
Post-Traumatic Stress	0.92	A short Post Traumatic Stress Disorder (PTSD) inventory (PTSD-8) [30] based upon the Harvard Trauma Questionnaire	A 4-point Likert scale was used to rate 8 items on whether participants were bothered by various symptoms directly linked to the Diagnostic and Statistics Manual of Disorders, fifth edition (DSM-IV) PTSD criteria. Scores were totaled per item with a score ≥3 on all of the 3 items (intrusion, avoidance or hypervigilance) indicative of PTSD symptomology.
Alcohol and Drug Use	0.91	Alcohol Use Disorder Identification Test (AUDIT) [31] shortened. Self-report drug and cigarette use	The types and frequency of alcohol, use was assessed. Items were used singly or scored for an indication of problematic drinking. Drug and cigarette use were assessed for type and frequency of use.
* Cronbach Alpha measured internal consistency of scales. A score of >70 is considered good			

#### Specimen collection and HIV testing

Following the survey, all participants were tested for HIV using two concurrently administered rapid finger prick tests, irrespective of self-reported HIV status (Table 4). All participants testing positive or indeterminate then received blood draws for further laboratory testing. Laboratory results (HIV Elisa, viral load, CD4 count) were provided to the partner organizations, who shared this information with all participants who returned to the site within 2 weeks of the data collection. Participants were given the opportunity to opt-out of this feedback process during informed consent, and, for those who opted out, results were not given to the partner organizations unless otherwise stipulated by the participant. All enrolled participants were issued with a letter confirming participation and referring them on for further ART or PrEP care (depending on the results of their HIV test during the study). This allowed them to seek medical care independently of the site’s sex work programme should they wish to do so. The laboratory barcode was scanned into the database and used to link survey and sample data. HIV drug resistance was done on all participants testing HIV positive with a viral load ≥400 copies/ml. Drug resistance results were not reported at the different sites as they were processed by the laboratory only after study completion.

**Table 4.** Rapid HIV test and other laboratory measures

VARIABLE NAME	MEASURE	DETAILS
HIV Status	2 concurrently run rapid assays	Two concurrent rapid finger prick assays were conducted to ascertain participants’ HIV status. The test kits used were the government-supplied screening and confirmatory test kits available at each site (Abon™, First Response®, Determine™, and Toyo test kits were all used). In the case of discordant rapid test results, blood samples were collected for a laboratory-based HIV enzyme-linked immunosorbent assay (ELISA).
Drug Resistance	In-house assay certified by the Virology Quality Assessment Program (VQA)	Sequencing of the pol gene were performed using an in-house assay certified by the Virology Quality Assessment Program (VQA). The procedure involves the generation of a nested Polymerase Chain Reaction (PCR) amplicon spanning the entire protease and p66 and p51 regions of the reverse transcriptase genes. Genotypic resistance was defined as the presence of resistance mutations associated with impaired drug susceptibility, using the Stanford University HIV Drug Resistance Database Calibrated Population Resistance Tool (http://cpr.stanford.edu/cpr.cgi) and the 2009 transmitted drug resistance mutation list. Phylogenetic analysis of nucleic sequences was performed using MEGA 5.05, and rates of transmission of resistance determined by calculating point prevalence with confidence intervals.
CD4 count	BD FACSCount™ system (TBC)	All blood samples for CD4 count were processed and performed by the local National Health Laboratory Service (NHLS) laboratory at the site of recruitment. The BD FACSCount system precisely measures absolute numbers and percentage results of CD4-positive T lymphocytes, the cellular parameter most closely associated with HIV/AIDS disease progression and therapy decisions. The system provides reproducible and accurate results even with low CD4 counts.
Viral Load	COBAS® AmpliPrep/COBAS® TaqMan® HIV-1 Test, v2.0(CAP/CTM) (TBC)	All blood samples for quantitative viral load measurement were processed and performed by the local NHLS laboratory at the site of recruitment. The dual-target COBAS® AmpliPrep/COBAS® TaqMan® HIV-1 Test, v2.0 enhances the reliability of HIV-1 viral load results and provides greater confidence while assessing disease and patient management. It is an in vitro nucleic acid amplification test for the quantitation of HIV-1 RNA in human plasma that targets two highly conserved regions of the HIV-1 genome, gag and long term repeat (LTR), not subject to drug pressure. In doing so, it compensates for the possibility of mutations or mismatches and increases the probability of detection. Dynamic range 20–1 000 000 copies/mL.
Recency Testing	Maxim HIV-1 Limiting Antigen Avidity EIA	Single well avidity enzyme immunoassay (EIA) for detection of recent HIV-1 infection.

### Data collection

Data were collected over a 7-month period from January to July 2019. Site data collection start dates were based on the district sample size as well as site readiness. We staggered site initiations to ensure that each site was prepared and had undergone a site readiness assessment by the core research team. This included a preparedness assessment, onsite training as required, and dry run sessions. Site-specific information was used to adapt recruitment in line with each sites’ dynamics, their staff and to ensure participant safety. Depending on the sample size, sites collected data over 2–5 months. At all but one site, data collection was implemented by a team of three: two SW counsellors/SW peer educators and one nurse. Due to the greater sample size (a factor of the density of the FSW population in the district), the Johannesburg site had three SW counsellors/SW peer educators and one nurse.

Informed consent, surveys and specimen collections were done in a private location either on or off-site, dependent on each organisation’s logistical requirements. Survey data collection was undertaken on a real-time basis using the REDCap mobile phone application and the data was centrally collated using the REDCap data management system [22]. At the end of each day, the interviewer uploaded the data to the REDCap server, which could then be viewed and checked by the core research team. Personal information collected directly into the REDCap data management system, was marked as ‘identifying’ to prevent the exportation of such information. Participant personal information was only accessible by approved study persons in the event of an emergency or to contact the participant for health-related matters. No personal information relating to their experiences of or perpetration of violence, or any other matter, was disclosed to any support service without express consent from the participant. Laboratory data was provided in separate spreadsheets and merged into the main database using each participant’s unique study identifier.

Data cleaning was conducted through identification of outliers for each variable, and appropriate adjustments were made where necessary. Data were reviewed by the core research team, and any errors were flagged and sent back to the site for rapid site feedback, and core team correction. This helped to ensure that site staff learnt how to improve their data collection techniques and to troubleshoot problems rapidly at the onset of the study. While we aimed for a 48-h turnaround time on database queries, technical challenges during data collection resulted in a delay to data uploads. Built-in skip patterns and algorithms were carefully developed to ensure ease of use and minimise human error.

**Figure 3.**
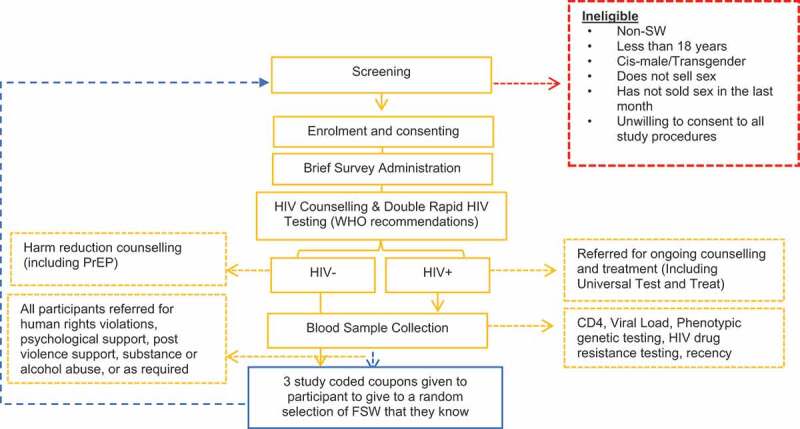
Flow of recruitment and data collection for FSWs

Pop-up alerts were coded into the database to help probe on more challenging aspects of the data collection. For example, multiple questions were asked about sexual violence. A pop-up alerted the interviewer to any inconsistencies in responses, which may have occurred from a capturing error, a misunderstanding, or due to a trauma response during this component of the interview, which required the interviewer to pause the interview and contain the participant. The interviewer could re-ask both questions if need be and respond as appropriate. Data completeness was high with a more than 95% completion rate; thus, we conducted a complete case analysis. Figure 3 details the flow of recruitment and data collection.

### Data analysis

Prior to statistical analysis, data were assessed for outliers that were cleaned through consultation with the research team and data collection study logs and in consultation with site study teams where necessary.

Frequencies and proportions (%) were determined for categorical variables overall and stratified by provinces. Medians and interquartile (IQR) ranges were determined for continuous variables and presented overall and by province. In the descriptive analysis presented in this manuscript, data were treated as a simple random sample illustrating the distribution of selected key variables. Despite the referral chain recruitment, we did not use RDS weighting because there were no overlapping networks between sites. The sample had no other weights applied as the study design was self-weighting in proportion to the provincial estimates of the size of the female sex worker population.

Statistical analysis was conducted using SAS Enterprise Guide 7.15 (SAS Institute Inc., Cary, NC, USA).

### Ethics approval

Prior to commencement, the study protocol, informed consent forms and survey tools were approved by the University of the Witwatersrand Human Research Ethics Committee (HREC) (Medical) (Ref number: 180,809).

## Results

The results presented in this section highlight the ability of the described study design to successfully and ethically enrol FSWs across multiple sites over a short period of time.

### Screening and enrolment

**Figure 4.**
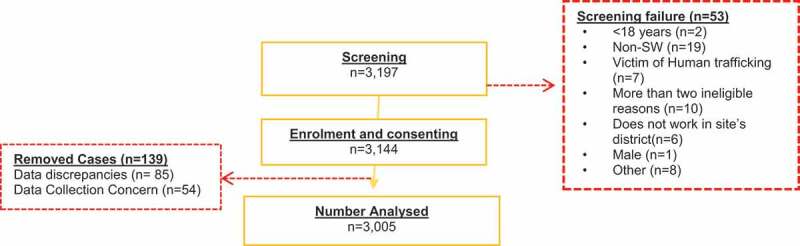
Disposition flow chart

A total of 3,197 FSWs were screened for inclusion across all 12 sites. Of these, 53 were ineligible for participation and 3,144 were enrolled in the study. Of those enrolled, 84 were removed due to data discrepancies (across multiple sites) as a result of a technical malfunction affecting REDCap data collection and another 54 were removed due to a data collection concern at one of the sites (Figure 4). Table 5 shows the sample size, which was achieved across all nine provinces within a period of 142 working days.

**Table 5.** Demographics, sexual behaviour and abuse data by province

**Variable**	**Overall (n = 3005)** **n (%)**	**Gauteng (n = 865)** **n (%)**	**North-West (n = 272)** **n (%)**	**Eastern Cape (n = 261)** **n (%)**	**Western Cape (n = 377)** **n (%)**	**Mpumalanga (n = 267)** **n (%)**	**KwaZulu-Natal (n = 431)** **n (%)**	**Northern Cape (n = 82)** **n (%)**	**Free State (n = 190)** **n (%)**	**Limpopo (n = 260)** **n (%)**
**Number of sites by province**	12	3	1	1	1	1	2	1	1	1
**Enrolment period in days**	142	100	104	97	120	99	127	65	105	73
**Demographic Characteristics**										
**Median age in years (Median, IQR)**	32.0 (27.0–38)	33.0 (28.0–39)	33.0 (29.0–39)	34.0 (28.0–41)	33.0 (28.0–37)	30.0 (26.0–36)	32.0 (27.0–38)	31.0 (26.0–35)	31.0 (25.0–35)	31.0 (25.0–38)
**Median age of first sex (Median, IQR)**	16.0 (15.0–18)	16.0 (15.0–18)	17.0 (15.0–18)	16.0 (15.0–18)	16.0 (15.0–17)	16.0 (15.0–18)	16.0 (14.0–17)	16.5 (15.0–19)	16.0 (15.0–17)	16.0 (15.0–18)
**First sexual experience**										
Non-consensual	718 (23.93)	194 (22.43)	79 (29.04)	55 (21.07)	72 (19.10)	86 (32.21)	146 (34.19)	23 (28.05)	31 (16.32)	32 (12.36)
Consensual	2282 (76.07)	671 (77.57)	193 (70.96)	206 (78.93)	305 (80.90)	181 (67.79)	281 (65.81)	59 (71.95)	159 (83.68)	227 (87.64)
**HIV Status**										
Indeterminate	6 (0.20)	1 (0.12)	1 (0.37)	0 (0.00)	0 (0.00)	1 (0.37)	1 (0.23)	0 (0.00)	2 (1.05)	0 (0.00)
Negative	1137 (37.84)	301 (34.80)	81 (29.78)	91 (34.87)	281 (74.54)	78 (29.21)	64 (14.85)	27 (32.93)	49 (25.79)	165 (63.46)
Positive	1862 (61.96)	563 (65.09)	190 (69.85)	170 (65.13)	96 (25.46)	188 (70.41)	366 (84.92)	55 (67.07)	139 (73.16)	95 (36.54)
**Sex work related**										
**Median years worked as sex worker (Median, IQR)**	6.0 (3.0–10)	5.0 (3.0–10)	5.0 (2.5–10)	5.0 (3.0–12)	7.0 (4.0–11)	5.0 (3.0–10)	7.5 (4.0–14)	5.0 (3.0–8)	5.0 (3.0–9)	5.0 (3.0–10)
**Outdoor versus Indoor based sex work**										
Street/outdoor-based	1137 (38.98)	183 (21.68)	41 (15.65)	123 (47.86)	113 (30.71)	134 (50.76)	341 (81.38)	26 (32.10)	149 (79.26)	27 (11.54)
Indoor/venue-based	976 (33.46)	526 (62.32)	132 (50.38)	86 (33.46)	62 (16.85)	48 (18.18)	18 (4.30)	4 (4.94)	23 (12.23)	77 (32.91)
Both outdoor and indoor	804 (27.56)	135 (16.00)	89 (33.97)	48 (18.68)	193 (52.45)	82 (31.06)	60 (14.32)	51 (62.96)	16 (8.51)	130 (55.56)
**Median number of clients in past day (Median, IQR)**	5.0 (3.0–8.0)	7.0 (4.0–10)	4.0 (2.0–7.0)	4.0 (3.0–6.0)	4.0 (3.0–7.0)	5.0 (3.0–8.0)	3.0 (2.0–5.0)	3.0 (2.0–5.0)	4.0 (3.0–5.0)	7.0 (4.0–10)
**Median earning potential per client (Median, IQR) (in ZAR)**	66.7 (44.3–106)	54.5 (38.9–77.8)	83.3 (50.0–100)	66.7 (42.9–117)	113 (50.0–183)	80.0 (50.0–110)	85.4 (50.0–125)	100 (50.0–158)	50.0 (40.0–70)	50.0 (28.6–100)
**Condom use in the past month**										
Consistent	551 (18.37)	117 (13.53)	50 (18.38)	26 (9.96)	116 (30.77)	57 (21.43)	83 (19.44)	27 (32.93)	68 (35.79)	7 (2.70)
Inconsistent	2448 (81.63)	748 (86.47)	222 (81.62)	235 (90.04)	261 (69.23)	209 (78.57)	344 (80.56)	55 (67.07)	122 (64.21)	252
**Violence Experienced (any)**										
Childhood abuse	2654 (88.44)	804 (92.95)	240 (88.24)	182 (69.73)	311 (82.49)	249 (93.61)	368 (85.98)	70 (85.37)	170 (89.47)	260 (100.0)
Intimate partner physical/sexual abuse in past 12 months	1376 (46.21)	339 (39.37)	91 (33.96)	104 (40.47)	176 (47.70)	132 (49.62)	215 (50.35)	27 (33.33)	60 (31.75)	232 (89.23)
Client physical/sexual abuse in past 12 months	1719 (57.38)	470 (54.34)	165 (60.89)	94 (36.02)	166 (44.15)	194 (73.21)	268 (62.62)	51 (62.20)	67 (35.26)	244 (94.57)
Police physical/sexual abuse in past 12 months	665 (22.25)	78 (9.07)	49 (18.08)	25 (9.65)	62 (16.45)	129 (49.05)	60 (14.02)	5 (6.10)	31 (16.32)	226 (7.2)
**Mental Health (any)**										
Depressive symptoms	1584 (52.71)	509 (58.84)	167 (61.40)	76 (29.12)	103 (27.32)	117 (43.82)	293 (67.98)	50 (60.98)	44 (23.16)	225 (86.54)
PTSD symptoms	1627 (54.14)	396 (45.78)	149 (54.78)	186 (71.26)	274 (72.68)	108 (40.45)	267 (61.95)	43 (52.44)	140 (73.68)	64 (24.62)

### Demographics, sexual behaviour and abuse data

The overall median age was 32 (IQR: 27–38) years, with the median age of first sex being 16 (IQR: 15–18) years, and 76.1% (n =2282/3000) of participants describing their first sexual experience as consensual. Overall, HIV prevalence was 62% (n =1862/3005) with the highest prevalence being in KwaZulu-Natal, Free State and Mpumalanga Provinces and the lowest in the Western Cape (Table 5).

Participants reported a median length of selling sex of 6 (IQR: 3–10) years, with over one-third sharing that they sell sex on the streets/outdoors (38.9%, n =1137/2917). KwaZulu-Natal and Free State had higher prevalences of selling sex on the street/outdoor compared to the other provinces. Participants had a median of 5 (IQR: 3–8) clients the day prior to enrolling in the study, with a median earning potential of ~5 (IQR: 3–8) USD per client. Western Cape and Northern Cape had the highest earning potential; and Gauteng, Free State and Limpopo province the lowest, valued at ~4 (IQR: 3–5) USD. Of all the participants, 81.6% (n =2448/2999) reported inconsistent condom use in the past month.

The majority of the participants reported experiencing childhood abuse (88.4%, n =2654/3001). Additionally, 46.2% (n =1376/2978) of participants had experienced physical/sexual abuse in the past 12 months from intimate partners, 57.4% (n =1719/2996) from clients and 22.3% (n =665/2989) from police. Limpopo Province had the highest prevalence of violence across all indicators. Over half of the participants reported having symptoms of depression (52.7%, n =1584/3005) and PTSD (54.1%, n =1627/3005).

## Discussion

This paper describes the development and implementation of the study through a community-centric approach, and offers lessons learned for research within FSW populations. Overall, our experiences of conducting the study suggest that implementing a nationwide survey with FSWs linked to sex worker organisations in South Africa and similar middle-income country settings is achievable, given active partnership with the sex worker organizations and collaborative engagement with SWs. Our overall screening failure percentage was only 1.7%, with 3% missing data. This is much lower compared to other studies that have reported screening failure rates of 12.9% [1]. We believe that engaging the sex work community and inviting them to be part of the study teams was fundamental to obtaining buy-in and commitment to the study. Our methodology was successful at accessing and appropriately enrolling FSW across multiple sites, with representation in all provinces, and across densely populated regions within the proposed study time period. Sample size targets were rapidly met within 6 months. This shows the importance of using a community-centric approach to studies with marginalised populations, such as SWs. Furthermore, the low percentage of missing data emphasises that with appropriate training and supportive technology, SW peer educators were able to successfully collect high-quality survey data.

Our study included an HIV rapid test, with blood draws for HIV positive or indeterminate participants. All participants agreed to be HIV tested and receive their results. The high uptake of HIV testing in this study highlights the importance of clearly articulating and explaining study procedures and the importance thereof to participants. This is especially important when the community being engaged is marginalised, and the research undertaken may result in positive programmatic changes. The study enabled the provision of site-specific feedback on the UN90:90:90 goals and gaps in service delivery (such as the need to strengthen mental health services), while also enabling linkage-to-care by study staff. Furthermore, HIVDR could be addressed for participants requiring regiment changes, and programme nursing staff engaged on the need to be vigilant about this within their programme.

As the first nationwide survey with FSW, our study highlights that the overall HIV prevalence of 62% is much higher than the 30.8% and 26.3% reported by women who attend antenatal clinics [5] and women aged 15–49 years [5], respectively. However, continued and additional funding is needed for existing programmes and for new programmes to be expanded into every district across South Africa. At the time of study initiation, there were only 22 sex worker programme sites across the 52 districts in South Africa, many of which have since closed due to the discontinuation of funding. The limiting of sites ultimately restricts the access to health services for SWs and undoes the hard work previously conducted by SWs themselves, sex work implementing partners, funders and government. This is particularly poignant given the impact that SARS-Cov-2 has had in disrupting access to HIV services [15], and the rapid rise in unemployment [32], which may result in an increase in sex-for-money transactions for survival.

The levels of violence reported in our national survey indicate that participants trusted the study teams and were enabled to disclose very personal experiences, often described in detail to the interviewers during the interview. Additionally, the violence results emphasise that FSWs are disproportionately exposed to trauma from childhood and continue to experience severe forms of violence into adulthood. Our study reported overall higher percentages of physical and sexual violence in the past 12 months (46.2% intimate partner, 57.2 client and 22.3% police) compared to 8% physical and 2% sexual violence amongst the general female adult population [33]. The overall high levels of lifetime and ongoing violence highlight the need to take a trauma-sensitive approach when conducting a project such as this, for both the participants and the study staff. It is imperative to ensure that the content of study questions and how they are asked is screened and approved by FSW prior to roll out. These initial steps facilitate the process of building trust while simultaneously ensuring that staff are supported to prevent secondary trauma and/or burnout during the study. Along with the counselling and trauma containment training provided as part of the study, staff members were also encouraged to partake in regular debriefing sessions, which were at times facilitated by an external member to prevent secondary trauma. Furthermore, the need for a social worker as a critical member of the study team, providing ongoing support to interviewers is paramount. While the violence figures highlight the vulnerability of FSW, they also indicate an urgent need for interventions focusing on male perpetrators. Evidence has shown that ~40% of men engage in some form of transactional or paid for sex [34]. The finding that >60% of new HIV infections are associated with men who pay for sex, highlights this massive gap in interventions and opportunity to intervene towards achieving the UN90:90:90 targets [6]. There are currently no programmes for high-risk men in South Africa, which the authors feel is a gross oversight in both addressing violence and HIV in the sex worker and general populations, and in supporting men to be part of the HIV and violence prevention solution.

We have managed to implement a rigorous study with a sample that approximates a random sample of sex workers in South Africa working in areas linked to programmes, and we have been able to collect interview data and specimens for biological analysis. We are not aware of a previously successful attempt to do this at a national level. Other strengths of the study included the efficient nature of the design to collect the required data and its ability to access a large sample size over a short period of time with a wide coverage and diversity across locations. Diversity is expressed across multiple levels, including the varying districts and venue types from which participants were enrolled. Furthermore, there is a high likelihood that the study results will be informative to policy makers. Part of the result of this study will be published in future publications. The study, however, also had a number of limitations. We cannot confirm the generalisability of our findings to health districts that do not have sex work programmes or to sex workers working outside the networks of established programmes. Several responses were self-reported and possibly influenced by social desirability bias, where respondents could have provided a more favourable answer to the interviewer. Additionally, while all efforts were made to limit inter-district enrolment and systems were developed to identify duplicates, there is no guarantee that participant was enrolled across two sites.

In summary, we have shown through this study that it is possible to conduct rigorous research with a very complex population engaged in illicit activities, such as sex work. The study methodology shows how this can be done in a way that is sensitive to the complexities of the population and scientifically rigorous. We suggest that programmes and research about sex workers, proactively engage the population from the conceptualisation phase of a project in order to maximise the benefits of the project for the population. The results from the national study provide policy makers with evidence to better understand the baseline prevalence of HIV, HIV incidence, HIVDR, adherence to ART or PrEP, violence, and mental ill health amongst FSWs.

## Author contributions

MM facilitated the design and oversaw the implementation and scientific aspects of the study and led the writing of the paper; RJ assisted with data analysis and interpretation and manuscript preparation and review; KO monitored and analyzed the data and contributed to writing; MJ oversaw the implementation of the medical component of the study and assisted with manuscript preparation and review; KH assisted with manuscript preparation and review; KH monitored and analyse the data and contributed to writing; MM assisted with study oversight and manuscript preparation; VM assisted with study oversight and manuscript preparation; GG provided technical and scientific oversight and manuscript review; KD assisted with data analysis and interpretation and manuscript preparation and review; GH provided scientific and laboratory oversight and contributed to review; AW assisted with data analyses and contributed to writing; RK assisted with data analyses and contributed to writing; NS technical input and manuscript review; LV technical input and manuscript review; AP laboratory oversight and review; AK technical input and review; NM technical oversight and manuscript review; FA provided technical and scientific oversight and manuscript review; JC oversaw the design, implementation and scientific aspects of the study and led the writing of the paper. All co-authors have provided their consent for publication

## Data Availability

The data that support the findings of this study are openly available in Mendeley Data at doi: 10.17632/hfr552s47v.1. https://data.mendeley.com/datasets/hfr552s47v/1

## Paper context

Research on the sex worker HIV epidemic in South Africa has primarily been conducted in metropolitan areas with a paucity of data describing the burden and vulnerabilities in less resourced settings. This manuscript presents the first national-level study designed to understand the HIV prevalence, incidence, violence, mental ill health, sexual and other risks, and treatment access among female sex workers in networks of programmes across the country.
